# Traumatic brain injuries in a district level emergency department in Cape Town: describing patients’ journey from arrival to CT scan and neurosurgery

**DOI:** 10.1186/s12873-025-01277-x

**Published:** 2025-07-15

**Authors:** Mignon du Toit, Daniël J van Hoving, Abigael Tamba, Ryan Mark O’Meara, Clint Hendrikse

**Affiliations:** 1 Division of Emergency Medicine, Department of Family and Emergency Medicine, Stellenbosch University, Cape Town, South Africa; 2 Division of Emergency Medicine, Department of Family, Community and Emergency Care, University of Cape Town, Cape Town, South Africa

**Keywords:** Traumatic brain injury, Head injury, Trauma, Low-and middle-income countries, Emergency medicine

## Abstract

**Background:**

Annually, 69 million people worldwide suffer a traumatic brain injury (TBI) with a disproportionately high prevalence of 500 to 800 per 100 000 population in low- and middle-income countries. Delays to accessing CT scan and neurosurgical services are associated with worse outcomes. This study describes the pathway from injury to CT scan and neurosurgery of patients with TBIs that presented to a district level emergency department (ED) in Cape Town.

**Methods:**

This was a retrospective cross-sectional analysis of patients presenting to a district level hospital in Cape Town and data were collected from existing databases and registries. All adult patients (≥ 13 years) who presented to the ED from 01 July 2021 till 30 June 2022 (12 months) with a suspected TBI and had a CT brain were eligible for inclusion. The description of the care pathway involved transfers to the nearby tertiary facility to access after-hour CT scan and neurosurgical services via interfacility transfers, as well as a description of emergency medical services (EMS) mission times for primary calls.

**Results:**

A total of 564 participants with TBIs were included in the final sample: *n* = 462 (82%) mild (GCS 13–15), *n* = 52 (9%) moderate (GCS 9–12) and *n* = 50 (9%) severe (GCS 3–8). Interpersonal violence accounted for more than half of injuries (*n* = 311, 55%), followed by traffic-related crashes (*n* = 115, 20%). For moderate and severe TBI, the median door-to-CT-scan time were 12 h and 9 h respectively, and door-to-neurosurgery time were 28 h and 20 h respectively. More than half (*n* = 305, 54%) of the CT scans were performed at the district facility, despite 73% (*n* = 410) of participants presenting outside of office hours. EMS transferred more than half (*n* = 302, 54%) of participants between the district and tertiary facilities.

**Conclusion:**

This study provides an understanding of patient journeys and pathway to access CT scan and neurosurgical services. Door-to-CT-scan time is much longer than what evidence suggest and relies heavily on interfacility transfers to access services. In particular, the pathway of the mild TBI cohort may require revision from a contextual best practice and limited resources point of view.

**Clinical trial:**

Not applicable.

**Supplementary Information:**

The online version contains supplementary material available at 10.1186/s12873-025-01277-x.

## Background

Globally, 4.4 million people die annually due to injuries and trauma [1]. In South Africa, injuries account for nearly 10% of all causes of mortality, with homicide and road traffic crashes being amongst the top 10 causes of mortality nationwide [2]. The burden of trauma in South Africa, particularly interpersonal violence, is significant and closely linked to alcohol misuse [3, 4]. For young people, those aged between five and 29 years, injuries account for three out of the top five causes of death, specifically road traffic crashes, homicide and suicide [1]. Despite the disproportionately high burden of trauma in low-and middle-income countries (LMICs), where 82% of the world’s population reside, best practice guidelines typically rely on data from high-income settings, often rendering them impractical, unachievable and unrealistic [5].

Despite advancements in healthcare, the long-term burden of non-fatal injuries on communities and families remain significant [6]. Not only is the trauma related burden disproportionately higher in LMICs, but outcomes are generally poorer and the economic impact more substantial. Road traffic crashes and injuries cost LMICs approximately 5% of the Gross Domestic Product, compared to 2% in high-income countries (HICs) [7]. The cost on families is often greater with the loss of income from people in their prime working years, cost of rehabilitation and ongoing medical care, plunging low-income families even further into poverty. Outcomes are generally poorer due to the added effect of inadequate healthcare systems and infrastructure and shortages of staff and consumables [5]. Furthermore, the non-fatal burden of injuries translate to an estimated 10% of all years lived with a disability [1]. 

Traumatic brain injury (TBI) is the leading cause of death and disability among all trauma-related injuries [8]. Annually, 69 million people worldwide suffer a TBI, with a disproportionately high prevalence in LMICs of between 500 and 800 per 100 000 population [8]. Furthermore, road traffic crashes account for 56% of these cases in both Africa and Southeast Asia, compared to only 25% in North America [8]. Trauma care pathways in LMICs are often not clearly defined, even though this is particularly important in settings where resources are limited or not available [5]. In HICs, patients with suspected TBI and a depressed level of consciousness often trigger a Trauma Team Activation pathway. This mobilises physical and human resources to streamline a patient’s flow through the emergency care pathway, improving outcomes by providing rapid access to Computed Tomography (CT) scan and neurosurgical services [9]. Without rapid access to CT scan and neurosurgical services, Vaca et al. (2019), from Uganda, reported that mortality rates for acute extradural and subdural haemorrhage doubled with delayed neurosurgical intervention [10]. 

The burden of trauma and interpersonal violence in South Africa is immense with trauma-related hospital visits accounting for 12 per 1000 population [11]. The timely diagnosis and appropriate management of suspected TBIs rely heavily on access to neuroimaging, i.e. CT brain scans, and access to neurosurgical services. In HICs, there are almost 40 CT scanners per million population compared to less than 1 per million in LMICs [12]. Furthermore, only 24% of the population in sub-Saharan Africa have access to a public facility with neurosurgical capabilities within a 2-hour window, compared to more than 93% in Europe [13]. Unfortunately, the paucity of local data hinders the development of contextually appropriate, pragmatic best practice guidelines, that takes into consideration resource availability and health seeking behaviour. Quantifying the burden and describing barriers to accessing CT and neurosurgery services are the first steps to address this health priority. We therefore aimed to describe the care pathway and access to CT-scan and neurosurgical services for adult patients with TBIs in a district level emergency department (ED) in Cape Town.

This study obtained approval from the Human Research Ethics Committee at Stellenbosch University (Ref S22/12/270) and the University of Cape Town (Ref 177/2023). Facility approval was obtained from the National Health Research Database (Ref: WC_202302_024) and from Groote Schuur Hospital.

## Methods

### Aim

To describe the care pathway and access to CT-scan and neurosurgical services for adult patients with TBIs in a district level ED in Cape Town.

### Study design

This was a retrospective cross-sectional analysis on data from existing databases and registries.

### Study setting

The study was conducted at Mitchells Plain Hospital (MPH) in Cape Town, South Africa, a large district (entry-level) hospital that serves a population of roughly 800 000 people. The ED attends to nearly 5 000 patients per month, overseen by four specialist emergency physicians. The hospital serves a community that comprises almost exclusively of low- to middle-income patients where drug abuse, crime, gangsterism, unemployment and poverty is rife [4]. At MPH, trauma accounts for 18% of ED visits with the remainder being for non-trauma related complaints [14]. 

The CT-scan service at MPH only operates during office hours (08:00 till 16:00 on weekdays) and patients requiring CT scans outside of office hours are transferred to a nearby tertiary hospital, Groote.

Schuur Hospital (GSH). Indications for CT scans in TBI are guided by the Western Cape Head Injury Guidelines (Supplementary data – Box 1) and are divided into two categories – those that require an immediate CT scan (within 1 h) and those that should get a CT scan within 8 h. Despite the high trauma burden, patients requiring specialist surgical care, such as neurosurgery, must be transferred to GSH [4]. Patients with TBIs requiring non-operative management are admitted to the district facility (MPH) for neurological observation, repeat CT scan or rehabilitation under the care of the general surgical department.

GSH is a tertiary academic hospital that is 22 km from MPH and receives referrals from several primary clinics, district and regional hospitals in Cape Town and the Western Cape. The facility boasts a level 1 trauma centre, 24-hour CT scan services, as well as specialised surgical services that include but is not limited to neurosurgery, ophthalmology, trauma surgery and maxillofacial surgery.

The decision to transfer patients for a CT scan or to await in-house imaging availability at the district facility is influenced by several factors, including anticipated EMS transfer delays, the current ED workload, and the likelihood of requiring neurosurgical intervention or intensive care—services not available at the district level.

### Population and sampling

This study employed a total population sampling approach. All patients who presented to the Mitchells Plain Hospital Emergency Department (ED) between 1 July 2021 and 30 June 2022 were screened for eligibility. The study focused on adult patients, defined as those aged 13 years or older within the South African healthcare system, who presented with a suspected traumatic brain injury (TBI) and underwent a CT brain scan during their ED visit.

#### Inclusion criteria

Patients were included if they were 13 years or older, presented to the ED with a suspected TBI, and received a CT brain scan during the index visit. Suspected TBI was identified based on initial clinical documentation in the ED, and the decision to perform CT imaging was guided by the Western Cape Head Injury Guidelines. Only CT scans that were requested by ED clinicians at the time of initial presentation were eligible for inclusion.

#### Exclusion criteria

Patients were excluded if clinical records or CT scan reports were incomplete or missing. Repeat CT scans linked to a prior injury episode were excluded, as were cases where the CT scan was ordered by an inpatient team rather than the ED. Patients whose injuries occurred during their hospital stay, as well as those whose CT scans could not be linked to the primary trauma episode, were also excluded. We excluded patients who succumbed from their head injury prior to arrival in the ED or those who died after arrival but prior to having a CT scan. Duplicate entries in the database were removed.

### Data collection and data management

Multiple data sources were accessed to describe the participants’ care pathway. Initially, the picture archiving system at the district hospital was utilised to identify eligible participants with a suspected TBI and a CT scan. The picture archiving system is used to acquire, store, transmit and display radiological images digitally, and were used to collect process times, CT scan reports and details with regards to the CT scan indication. The hospital’s digital patient management application was utilised to capture demographic details, ED disposition and process times. All participants that were transferred to the tertiary hospital (for CT-scan or neurosurgery) were also identified. The picture archiving system at the tertiary hospital were used to collate these participants’ CT scan reports and CT processing times.

Clinical details, such as the Glasgow Coma Score (GCS), mechanism of injury and neurosurgical intervention, were obtained from patient records. Participants who were transferred with public Emergency Medical Services (EMS), whether primarily or between facilities, were identified and prehospital data were obtained from the electronic pre-hospital database which include process times, and clinical and geospatial data. A detailed explanation of all the time variables can be found in the supplementary material in Box 2. All applications utilised are official Western Cape Department of Health and Wellness provincial applications and the Patient Identifier Number was used to access information.

All study investigators and data collectors were health care professionals and familiar with the databases and registries. The data collection processes, case selection, definitions and the data collection sheet were standardised, and all investigators were trained. Data from a random 5% of the sample were independently reviewed by two investigators and cross checked to monitor the accuracy and consistency of the data. Discrepancies were discussed and resolved through consensus.

#### Data safety and monitoring

Patient Identifier Numbers were the only identifier collected to track the participants through the various databases and registries. Data were pseudo-anonymised once the data-collection process was concluded. The Patient Identifier Numbers were then saved against unique study numbers in a separate file in cases of a query. Only the principal investigator has access to this password protected file.

#### Data analysis

Data were analysed using IBM SPSS Statistics for Windows, Version 29.0.2.0 (IBM Corp., Armonk, NY). Only descriptive statistics were performed. Categorical variables were summarised using frequencies and percentages. Continuous variables were described using medians and interquartile ranges (IQR), as the data were not normally distributed. No inferential statistical testing was undertaken, as the aim of the study was to provide a descriptive overview of patient characteristics and care pathways. TBIs were classified using the GCS, as mild (GCS of 13–15), moderate (GCS 9–12) and severe (GCS ≤ 8) [15, 16].

## Results

**Fig. 1:**
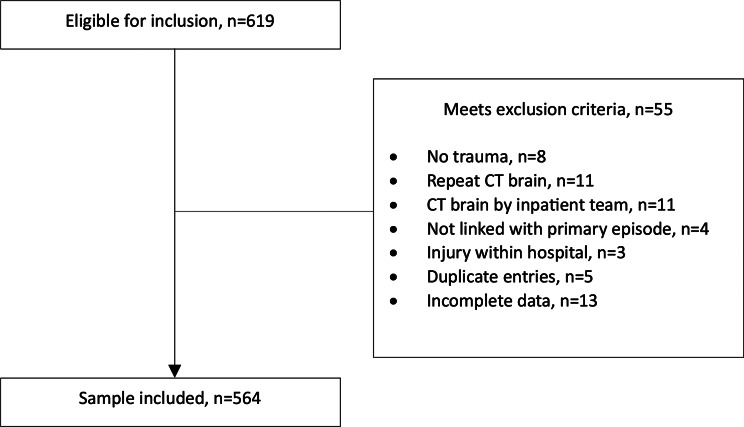
Flow diagram of study participants. CT Computed Tomography

A total of 564 participants were analysed after 55 patients were excluded (Fig. 1).

The sample comprised of 462 (82%) mild TBI (GCS 13–15), 52 (9%) moderate TBI (GCS 9–12) and 50 (9%) severe TBI (GCS 3–8) cases. Male participants (*n* = 452, 80%) predominated the sample overall and were even more prevalent in the moderate (*n* = 46, 89%,) and severe TBI categories (*n* = 47, 94%). The age distribution was skewed to the right (median 32, IQR 19 years) with 439 (78%) being younger than 45 years old (Table 1). Participants who self-presented with private transport were more prevalent in the mild TBI category (*n* = 252, 57%), while participants arriving directly from scene with EMS (public and private) were more prevalent in the moderate (*n* = 30, 58%) and severe (*n* = 28, 56%) categories. Most participants arrived outside of office hours (*n* = 410, 73%) with Sundays being the most prevalent day of presentation to the ED (*n* = 120, 21%). One in three participants arrived on weekends (*n* = 199, 35%). Interpersonal violence (*n* = 311, 55%) was the most prevalent category of injury, with road traffic related (*n* = 115, 20%) and accidental injuries (*n* = 108, 19%) comprising the bulk of the rest (Table 1). The prevalence of road traffic crashes did however increase with the more severe TBI categories (*n* = 15, 30%), while the prevalence of accidental injuries decreased (*n* = 1, 2%). Blunt assault was the predominant mechanism of injury in all three TBI categories accounting for 211 (46%), cases of mild TBI, 18 (35%) cases of moderate TBI and 25 (50%) of severe TBIs. Pedestrian motor vehicle crashes were the second most prevalent cause of severe (*n* = 12, 24%) and moderate (*n* = 7, 14%) TBI (Table 1).

**Table 1:** Demographics, presenting characteristics and injury mechanism of participants with traumatic brain injuries

*n* (column%)	Overall *n* = 564	Traumatic brain injury category		
		GCS 13–15 *n* = 462	GCS 9–12 *n* = 52	GCS 3–8 *n* = 50
Age category				
≤ 25 years	166 (29.4%)	131 (28.4%)	14 (26.9%)	21 (42.0%)
26–45 years	273 (48.4%)	228 (49.4%)	24 (46.2%)	21 (42.0%)
46–65 years	79 (14.0%)	62 (13.4%)	10 (19.2%)	7 (14.0%)
> 65 years	45 (8.0%)	40 (8.7%)	4 (7.7%)	1 (2.0%)
Gender				
Male	452 (80.1%)	359 (77.7%)	46 (88.5%)	47 (94.0%)
Female	112 (19.9%)	103 (22.3%)	6 (11.5%)	3 (6.0%)
Referral type				
Walk-ins	270 (47.9%)	228 (49.4%)	20 (38.5%)	22 (44.0%)
Primary EMS	203 (36.0%)	153 (33.1%)	23 (44.2%)	27 (54.0%)
Primary health care	58 (10.3%)	50 (10.8%)	7 (13.5%)	1 (2.0%)
General practitioner	26 (4.6%)	25 (5.4%)	1 (1.9%)	0
South African Police Service	5 (0.9%)	4 (0.9%)	1 (1.9%)	0
Non-study district ED	2 (0.4%)	2 (0.4%)	0	0
Transport				
Private transport	305 (54.1%)	262 (56.7%)	21 (40.4%)	22 (44.0%)
Public EMS	248 (44.0%)	191 (41.3%)	30 (57.7%)	27 (54.0%)
Private EMS	6 (1.1%)	5 (1.1%)	0	1 (2.0%)
South African Police Service	5 (0.9%)	4 (0.9%)	1 (1.9%)	0
Day of arrival				
Monday	88 (15.6%)	70 (15.2%)	10 (19.2%)	8 (16.0%)
Tuesday	69 (12.2%)	57 (12.3%)	5 (9.6%)	7 (14.0%)
Wednesday	68 (12.1%)	62 (13.4%)	4 (7.7%)	2 (4.0%)
Thursday	66 (11.7%)	53 (11.5%)	9 (17.3%)	4 (8.0%)
Friday	74 (13.1%)	60 (13.0%)	6 (11.5%)	8 (16.0%)
Saturday	79 (14.0%)	62 (13.4%)	8 (15.4%)	9 (18.0%)
Sunday	120 (21.3%)	98 (21.2%)	10 (19.2%)	12 (24.0%)
Weekend				
Yes	199 (35.3%)	160 (33.6%)	18 (34.6%)	21 (42.0%)
No	365 (64.7%)	302 (65.4%)	34 (65.4%)	29 (58.0%)
Office hours				
Yes	154 (27.3%)	131 (28.4%)	12 (23.1%)	11 (22.0%)
No	410 (72.7%)	331 (71.6%)	40 (76.9%)	39 (78.0%)
Injury category				
Interpersonal violence	311 (55.1%)	254 (55.0%)	26 (50.0%)	31 (62.0%)
Road traffic related	115 (20.4%)	89 (19.3%)	11 (21.2%)	15 (30.0%)
Accidental	108 (19.1%)	97 (21.0%)	10 (19.2%)	1 (2.0%)
Unknown	30 (5.3%)	22 (4.8%)	5 (9.6%)	3 (6.0%)
Mechanism/cause of injury Accidental				
Fall – mechanical	84 (14.9%)	76 (16.5%)	7 (14%)	1 (2.0%)
Fall – medical (syncope)	20 (3.5%)	18 (3.9%)	2 (4%)	0
Sports related	4 (0.7%)	3 (0.6%)	1 (2%)	0
Interpersonal Violence				
Blunt assault	254 (45.0%)	211 (45.7%)	18 (34.6%)	25 (50.0%)
Penetrating assault	38 (6.7%)	32 (6.9%)	5 (9.6%)	1 (2.0%)
Firearm injury	15 (2.7%)	9 (1.9%)	2 (3.8%)	4 (8.0%)
Self-inflicted	4 (0.7%)	2 (0.4%)	1 (1.9%)	1 (2.0%)
Road traffic related				
Pedestrian vehicle collision	67 (11.9%)	48 (10.4%)	7 (13.5%)	12 (24.0%)
Motor vehicle crash	42 (7.4%)	35 (7.6%)	4 (7.7%)	3 (6.0%)
Bicycle crash	4 (0.7%)	4 (0.9%)	0	0
Motorcycle crash	2 (0.4%)	2 (0.4%)	0	0
Unknown				
Unknown	30 (5.3%)	22 (4.8%)	5 (9.6%)	3 (6.0%)

GCS Glasgow Coma Scale, EMS Emergency Medical Services. Percentages may not always add up to 100% due to rounding

A total of 564 CT scans were performed across both hospitals (Table 2). Of these, 305 (54%) CT scans were obtained at the district level hospital and 259 (46% at tertiary level). More than half (*n* = 302, 54%) of all participants were transferred to the tertiary hospital, the majority for CT scan (*n* = 259), and a smaller proportion (*n* = 40) as referrals to neurosurgery (participants who had their CT scan at the district level hospital). A total of 434 (77%) CT scans were reported with pathology. Of these, 209 (37%) revealed intracranial blood with/without skull fractures, 159 (28%) showed extracranial facial or other injuries not related to the calvarium and/or brain, 53 (9%) had only old/existing changes, and 13 (2%) revealed acute medical pathology not related to trauma. Overall, 42 participants (14%) required neurosurgery. Intensive care was required for 31 (6%) participants, and 24 (4%) were nursed in high-care units. From the district hospital ED, 134 (24%) participants were admitted and 123 (22%) were discharged. Of those transferred to the tertiary hospital for CT scan, 74% (*n* = 220) were discharged home, either from the trauma unit or from the ward, and 16% (*n* = 48) were transferred back to the district hospital for further treatment. Most participants (*n* = 465, 83%) were triaged Red (emergency) or Orange (very urgent) per the South African Triage Scale (Table 2) [17]. Among patients with severe TBI, 30 (60%) were triaged Red, 47 (94%) were intubated (mostly at the district hospital), and 10 (20%) received oxygen without ventilation. In-hospital mortality was 8% (*n* = 26), with the majority of deaths (*n* = 17) occurring in patients with severe TBI. (Table 2).

**Table 2:** Clinical characteristics and treatment requirement of participants with traumatic brain injury

*n* (column%)	Overall *n* = 564	Traumatic brain injury category		
		GCS 13–15 *n* = 462	GCS 9–12 *n* = 52	GCS 3–8 *n* = 50
Patient acuity^#^				
Emergency (Red)	89 (15.8%)	46 (10.0%)	13 (25.0%)	30 (60.0%)
Very urgent (Orange)	376 (66.7%)	321 (69.5%)	35 (67.3%)	20 (40.0%)
Urgent (Yellow)	95 (16.8%)	91 (19.7%)	4 (7.7%)	0
Routine (Green)	4 (0.7%)	4 (0.9%)	0	0
Oxygen received				
Yes	28 (5.0%)	11 (2.4%)	7 (13.5%)	10 (20.0%)
No	536 (95.0%)	451 (97.6%)	45 (86.5%)	40 (80.0%)
Intubated				
Yes	77 (13.7%)	16 (3.5%)	14 (26.9%)	47 (94.0%)
No	487 (86.3%)	446 (96.5%)	38 (73.1%)	3 (6.0%)
Intubated prehospitally	1 (0.6%)	0	1 (7.1%)	0
Intubated at district facility	56 (9.9%)	6 (37.5%)	6 (42.9%)	44 (93.6%)
Intubated at tertiary facility	20 (7.8%)	10 (62.5%)	7 (50.0%)	3 (6.4%)
Location where CT scan performed				
District facility	305 (54.1%)	265 (57.4%)	25 (48.1%)	15 (30.0%)
Tertiary facility	259 (45.9%)	197 (42.6%)	27 (51.9%)	35 (70.0%)
Door-to-CT-scan (proportion)*				
< 1 h	13 (2.3%)	7 (1.5%)	0	6 (12.0%)
< 8 h	230 (40.8%)	171 (37.0%)	22 (42.3%)	37 (74.0%)
CT scan report				
Intracranial blood with/without skull fracture	209 (37.1%)	143 (31.0%)	30 (57.7%)	36 (72.0%)
Extra cranial injuries and fractures only	89 (15.8%)	77 (16.7%)	6 (11.5%)	6 (12.0%)
Soft tissue injury only	70 (12.4%)	64 (13.9%)	4 (7.7%)	2 (4.0%)
Acute pathology (not trauma related)	13 (2.3%)	10 (2.2%)	3 (5.8%)	0
Old/existing changes only	53 (9.4%)	51 (11.0%)	2 (3.8%)	0
Normal	130 (23.0%)	117 (25.3%)	7 (23.5%)	6 (12.0%)
District level ED disposition				
Transferred out to tertiary care	302 (53.5%)	222 (48.0%)	34 (65.4%)	46 (92.0%)
For CT brain	259 (45.9%)	197 (42.6%)	27 (51.9%)	35 (70.0%)
For neurosurgery	40 (7.1%)	22 (4.8%)	7 (13.5%)	11 (22.0%)
For ophthalmology or orthopaedics	3 (0.5%)	3 (0.6%)	0	0
Admission	134 (23.8%)	116 (25.1%)	14 (26.9%)	4 (8.0%)
Discharged	123 (21.8%)	120 (26.0%)	3 (5.8%)	0
Left before completion of care	4 (0.7%)	4 (0.9%)	0	0
Deceased	1 (0.2%)	0	1 (1.9%)	0
Underwent neurosurgery				
Yes	42 (14.2%)	24 (11%)	8 (23.5%)	10 (22.7%)
No	253 (85.8%)	193 (90%)	26 (76.5%)	34 (77.3%)
Admitted to ICU				
Yes	31 (5.5%)	18 (3.9%)	3 (5.8%)	10 (20.0%)
No	533 (94.5%)	444 (96.1%)	49 (94.2%)	40 (80.0%)
Admitted to HCU				
Yes	24 (4.3%)	17 (3.7%)	2 (3.8%)	5 (10.0%)
No	540 (95.7%)	445 (96.3%)	50 (96.2%)	45 (90.0%)
Tertiary care disposition				
Discharged	220 (73.8%)	175 (79.5%)	23 (67.6%)	22 (50.0%)
Transferred back to district level facility (stepdown)	48 (16.1%)	39 (17.7%)	5 (14.7%)	4 (9.1%)
Deceased	25 (8.4%)	3 (1.4%)	5 (14.7%)	17 (38.6%)
Transferred to rehabilitation centre	5 (1.7%)	3 (1.4%)	1 (2.9%)	1 (2.3%)
In-hospital mortality	26 (4.6%)	3 (0.6%)	6 (11.5%)	17 (34.0%)

^#^According to the South African Triage Scale, * Thresholds from Western Cape Head Injury Guideline (Supplementary material – Box 1)

GCS Glasgow Coma Scale, CT Computed Tomography,

ICU Intensive Care Unit HCU High Care Unit ED Emergency department. Percentages may not always add up to 100% due to rounding

Of those who arrived via public EMS from the scene, the median mission time was 1 h and 23 min (Table 3). A detailed explanation of the time variables can be found in the supplementary material in Box 2. Similar process times were observed for all categories of TBI with minor discrepancies, including longer scene times for the moderate TBI category. The total length of stay in the ED was 15 h and 8 min overall but was considerably shorter (9 h and 18 min) for those in the severe TBI category. A door-to-CT-scan time of 13 h and 32 min overall were observed with a marked reduction in the severe TBI category (8 h and 42 min). Similarly, an overall door-to-neurosurgery time of 31 h and 21 min were observed with a marked reduction in the severe TBI category (19 h 30 min). It is important to note that the time intervals reported in Table 3 represent median values calculated independently for each phase of care (e.g., door-to-triage, triage-to-consultation, etc.). These medians are not derived from the same individual patient trajectories, and as such, the sum of these medians does not equate to the overall median Emergency Centre (EC) length of stay. This is an expected statistical outcome, particularly in the presence of skewed distributions, which are common in operational time data.

**Table 3:** Prehospital and in-hospital process times for all participants with traumatic brain injury

Hours: minutes (median, Q1-Q3)	Overall *n* = 564	Traumatic brain injury category		
		GCS 13–15 *n* = 462	GCS 9–12 *n* = 52	GCS 3–8 *n* = 50
Pre-hospital (*n* = 248)				
Response time	00:30 (00:17–01:00)	00:29 (00:17–00:56)	00:45 (00:20–01:32)	00:28 (00:16–01:14)
Scene time	00:18 (00:10–00:28)	00:18 (00:11–00:27)	00:23 (00:09–00:28)	00:16 (00:08–00:36)
Transportation time	00:27 (00:15–00:37)	00:28 (00:15–00:38)	00:18 (00:07–00:30)	00:29 (00:23–00:37)
Total mission time	01:23 (01:05–01:57)	01:24 (01:07–01:50)	01:19 (00:23–04:42)	01:28 (01:00–02:08)
District facility ED (*n* = 564)				
Door to triage	00:19 (00:06–00:47)	00:21 (00:07–00:48)	00:11 (00:03–00:58)	00:10 (00:03–00:22)
Triage to consultation	02:08 (00:32–05:21)	02:32 (00:40–05:54)	01:19 (00:23–04:42)	00:28 (00:04–01:28)
Consultation to ED disposition	05:31 (02:25–11:06)	05:52 (02:45–11:16)	05:54 (01:46–11:16)	03:13 (01:36–07:24)
ED disposition to ED exit	04:03 (01:35–07:13)	04:02 (01:35–07:30)	04:24 (01:46–07:59)	02:45 (01:15–05:42)
Total ED length of stay	15:08 (09:25–23:04)	15:51 (10:07–24:04)	15:01 (09:47–19:28)	09:18 (04:29–14:44)
Door to CT scan	13:32 (08:02–18:10)	14:14 (08:44–18:37)	11:29 (08:36–16:40)	08:42 (05:34–12:27)
Door to neurosurgery	31:21 (20:11–58:25)	42:07 (28:49–71:21)	28:18 (19:50–40:11)	19:30 (14:04–38:43)

Q1 25th percentile Q3 75th percentile GCS Glasgow Coma Scale

ED Emergency Department CT Computed Tomography

**Fig. 2:**
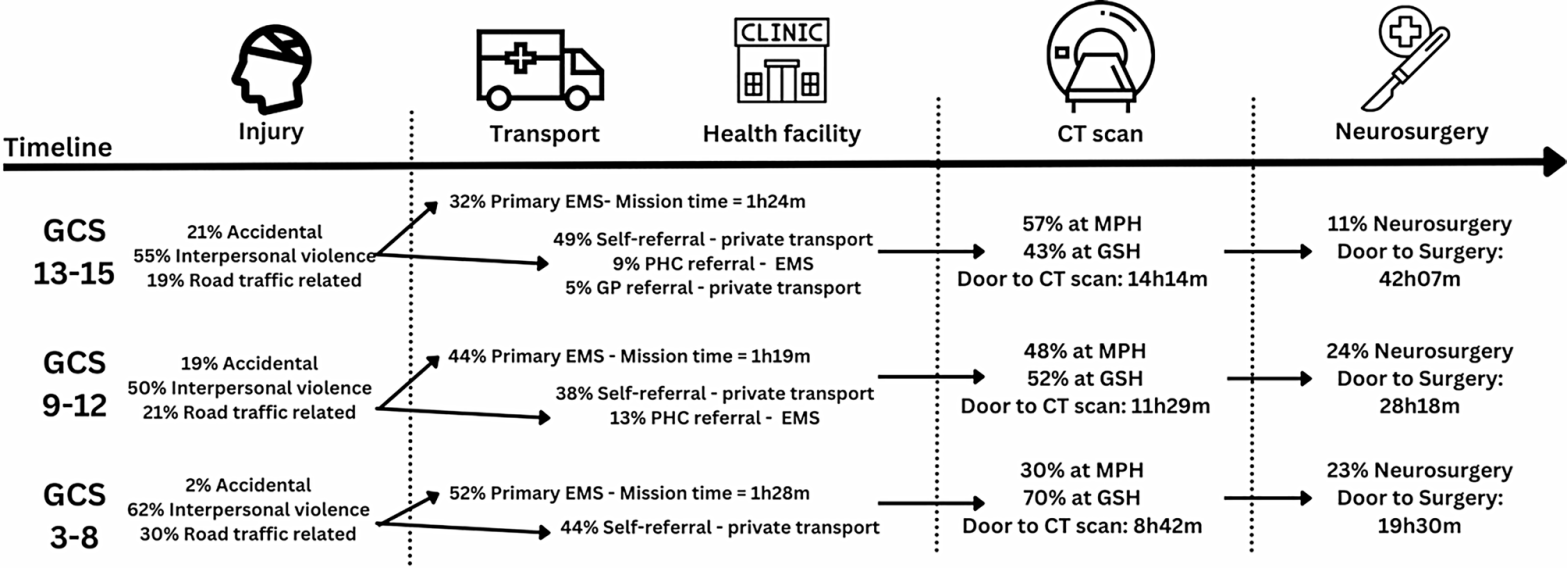
Pathway to CT scan and neurosurgery for patients with traumatic brain injuries. CT Computed Tomography GCS Glasgow Coma Scale EMS Emergency Medical Services PHC Primary Health Care. MPH Mitchells Plain Hospital GSH Groote Schuur Hospital

Fig. 2 summarises the care pathway of patients with TBI from time of activating the health system to obtaining a CT scan or neurosurgery.

## Discussion

Participants with TBIs were mostly men under the age of 45 years, with interpersonal violence, and pedestrians involved in vehicular incidents were the most frequent mechanism. We described a care pathway that is heavily reliant on EMS to transfer patients to access CT-scan and neurosurgical services. The median door-to-CT-scan and door-to-neurosurgery time was around 14 h and 31 h respectively. Despite almost half of participants being transferred from district to tertiary level for CT scan, many these patients were discharged after CT scan and very few had neurosurgical interventions. Services related to EMS, CT and neurosurgery are all limited resources that should be prioritised for patients that will benefit the most to ensure that the time from door-to-neurosurgery is minimised.

This profile of TBI was characterised by young male patients, primarily resulting from interpersonal violence and pedestrian injuries with a predilection for weekends. Males comprised 80% of the sample, and 78% of the participants were younger than 45 years old. This pattern is echoed in studies from Uganda and Botswana, where males accounted for between 69% and 86% of TBIs and more than 80% of participants were under the age of 40 years [10, 18, 19]. This is notable, as this demographic typically represents a significant portion of the economically active population and often holds key roles in providing for their families. Preventative strategies should thus continue to focus on this high-risk group.

Interpersonal violence was the most prevalent mechanism of injury, with 55% of all TBIs attributed to assault. This contradicts patterns from HICs where falls account for 56% of TBIs [10]. It is also different to other African countries, specifically Botswana, Ethiopia and Uganda, where road traffic related (vehicular) injuries were the most prevalent mechanism of injury of TBIs (38 − 62%) [18–20]. Interestingly, pedestrian injuries were the third most prevalent mechanism of injury in our setting, more than other vehicular related injuries, especially in the moderate and severe cohort. The Road Traffic Management Corporation of South Africa reported that in 2023, pedestrians accounted for 45% of all vehicular fatalities [21]. Only one study from Ethiopia reflected a similar pattern where pedestrian vehicle crashes accounted for 52% of TBIs resulting from vehicular accidents [20]. This TBI profile can probably be linked to underlying factors such as alcohol abuse and prevailing social norms. Alcohol consumption, particularly in social contexts, has been well-documented as a contributing factor in both interpersonal violence and road-related injuries [22, 23]. The effects of alcohol on injuries and specifically interpersonal violence was perhaps best demonstrated during the full alcohol ban as part of the lockdown regulations of the COVID-19 pandemic in South Africa. ED presentations with injuries and trauma decreased by 19% during periods with no alcohol sales [4]. Govender et al. (2021) reported that between 2016 and 2018, alcohol-related RTCs accounted for between 33% and 69% of crashes [24]. 

The prolonged door-to-CT-scan times observed in this study (median = 13h32), underscore the significant delays compared to the recommendations by the Western Cape Head Injury Guidelines. Even though the median times were reduced in the moderate (11h29m) and the severe TBI categories (08h42m), they remain much longer than what is recommended [14]. Schellenberg et al. (2021) illustrated that immediate CT brain (< 1 h) resulted in a faster ED throughput and shorter time to neurosurgical intervention, but comparing data and recommendations from the United States to a middle-income country like South Africa, where CT scan services remains a limited resource, is challenging without contextualisation [9]. In this study performed in the USA, the median time to CT scan was 0.5 h for an “immediate” CT scan and 1.6 h for a “delayed” CT scan, making it almost impossible to compare these time variables with LMICs [25]. Clinical decision rules and clinical guidelines from HICs aims at reducing risk as much as possible by leaning towards a higher sensitivity, thereby accepting a high false negative rate. Our study did not assess strict adherence to the Western Cape Head Injury Guidelines. However, the high number of mild TBI cases transferred for imaging suggests a need to evaluate whether the current criteria are sufficiently specific in resource-constrained environments.

In LMICs, guidelines and clinical decision rules are often created around the availability of limited resources and tends to lean towards a higher specificity, allowing less false positives [26–28]. In HICs there are nearly 40 CT scan machines and nearly 100 radiologists and radiographers per million population compared to middle income countries where only one machine and 1.9 radiologists and radiographers per million population exist [29, 30]. In South Africa, access to CT scan services is further complicated by the marked discrepancy in CT availability between the public and private sector, as well as inter-provincial variability [25, 31]. The majority of the South African population (83%) utilises the public health service that has access to 5 CT machines per million population, 5 times the African average. The remaining 17% of the population has access to private medical services that boasts 21 CT machines per million population [29, 31]. Kabongo et al. (2015) highlights that there is a 10-fold variation in the availability of CT-scans across provinces [31]. The inequitable access to imaging technology in South Africa potentially contributes to the delays in the delayed door-to-CT scan times.

The utilisation of EMS plays a key role in enabling access to CT imaging and neurosurgical services, for both primary transport and interfacility transfers. In this cohort, 44% of participants arrived at the district level facility via EMS, and an additional 54% were subsequently transferred to the tertiary facility for CT scan or neurosurgical evaluation. Additionally, 18% were later transferred from the tertiary facility to stepdown or rehabilitation facilities, suggesting that some patients required up to three EMS transfers during a single TBI admission. Notably, 46% of the cohort required transfer specifically for a CT scan, yet 74% were ultimately discharged home from the tertiary hospital – either directly from the ED or after an inpatient stay– including 80% of those with mild TBI. While this does not necessarily imply that these patients should not have been transferred, the relatively low proportion requiring Intensive care/high care admission or neurosurgical intervention (as shown in Table 3) raises important questions about the efficient of the current care pathway.

In a resource challenged setting with constrained EMS availability and restricted access to CT imaging, these findings suggest that the existing system may benefit from re-evaluation – particularly in identifying patients with mild TBI who may be safely observed or scanned during routine hours at the district level. One option is to upscale the CT services at district hospitals, which would carry additional costs but could be offset by reduced EMS transfers. Alternatively, selected patients with less severe TBI could remain at the district level until CT becomes available. However, the impact of such delays on patient centred outcomes remains uncertain. Schellenberg et al. (2021), demonstrated that a shorter door-to-CT-scan time resulted in a shorter door-to-neurosurgery time in those with a moderately depressed GCS, but did not demonstrate a reduced mortality or reduced need for craniotomy among patients undergoing immediate CT scan, i.e. within 1 h [9]. Another consideration is to relook at guidelines indicating CT brain for TBIs to attempt to limit unnecessary scans. Emerging tools, such as plasma-based biomarkers, which reports negative predictive values above 99%, may offer future promise for streamlining in low-resource settings by reducing unnecessary imaging [32]. Regardless of which option is preferred, a bird’s-eye approach is needed to create an optimal cost-effective system without compromising patient safety [33]. 

Significant delays in the overall door-to-neurosurgery time (median 31 h) were encountered. A Malaysian study demonstrated a median door-to-skin time of 10 h and established that delays were strongly associated with poorer outcomes [34]. Furthermore, one in four participants in the current study with moderate and severe TBIs required neurosurgery – with a door-to-skin time of around 20 h. This operative rate is higher than the approximately 16% reported for moderate and severe TBIs by Vaca et al. (2019) in Uganda. Sweeney et al. (2015) report an 8.8% operative rate for those patients with a mild TBI, compared to the 11% in our population [24]. Unfortunately, selection bias likely precludes any meaningful comparisons as each hospital, and country, offered different levels of care to different patient profiles [10]. In resource limited settings, prioritising CT scans for moderate and severe TBI patients could expedite time-to-skin for those requiring surgery, while a more conservative approach for mild TBI may be appropriate to achieve best outcomes for the most patients.

The typical patient care pathway as illustrated in Fig. 2, highlights key delays and the frequent need for transfers between the district and tertiary facility. An assessment of the care pathway and specifically involving the whole health ecosystem, is essential to understand how patients access acute care, as well as understanding where the bottlenecks in the system occur. Even though this is aligned with the World Health Organisation’s integrated emergency, critical and operative (ECO) framework, as well as their emergency care framework, collecting the necessary data remains challenging in LMICs with absent or nascent information systems [35]. While this study demonstrates the value of a longitudinal assessment of a care pathway, significant investment is required to overcome these barriers [36–38]. 

### Limitations

Even though this retrospective study assessed a care pathway in one health ecosystem in Cape Town, the authors believe that the results are representative of the rest of Cape Town and perhaps health systems with similar resource challenges. The pathway description excluded patients that were transported directly from the scene to the tertiary hospital, as well as patients that were transported from other facilities to the tertiary hospital. Although impossible to confirm, patients transported directly by EMS from scene to a tertiary facility, are likely assessed to be more severely injured than those that are taken to the district-level hospital. A comprehensive understanding of how patients with TBIs access the health system within the broader health ecosystem remains unknown.

Our sample only included participants who had a CT brain – possibly excluding patients with mild TBIs. Patients with severe TBIs that succumbed prior to hospital arrival or in the ED prior to having a CT scan were also not included. Selection bias in these scenarios could therefore have both under- and overestimated our results. Trauma severity was not measured, and this may have a confounded effects on those patients with polytrauma. We decided to use the traditional and widely used GCS categorisation of TBIs, even though it is not very practical or helpful as it does not differentiate or predict outcomes or the need for interventions [39]. Although the time-to neurosurgery was reported, it may have been insightful to also report the time to neurosurgery consultation, as there are often many factors that may cause delays from the time of neurosurgery consultation to eventual surgery.

### Future studies

Future studies should be multi-centred and include patients’ journey throughout the health system from the time of injury to fully understand the burden of TBIs in Cape Town. Establishing and investing in a TBI registry would provide sustainable good quality data that can inform future practise and help establish evidence-based guidelines. The three-delays framework should be explored to identify delays to CT and neurosurgery services in order to identify ways to expedite care for the most appropriate patients [40]. Furthermore, to better determine resource allocation, establishing a practical outcome based guideline for patients with TBIs may be beneficial [41]. This study contributes to ongoing work aimed at understanding the application of the Western Cape Head Injury Guidelines and informing improvements in access to after-hours CT imaging, which is often limited by EMS availability and other system constraints.

## Conclusion

The pathway to care for patients with TBI in Cape Town is convoluted and system-centred around limited resources. Patients’ journey through the health system is prolonged and heavily dependent on EMS to help facilitate interfacility referrals with substantial delays to accessing CT-scan and neurosurgical services. These challenges are likely a reflection of similar pathways in other LMICs. Best practice guidelines can therefore not be adopted from high-income settings but should be context specific and aimed at utilising the limited resource to provide the best impact. The study highlights several areas for further investigation. The longitudinal pathway description provided insight into patients’ journey from time of arrival through the complex emergency care system and generated hypotheses for further investigation.

## Electronic supplementary material

Below is the link to the electronic supplementary material.

Supplementary Material 1

## Data Availability

Data is provided within the manuscript or supplementary information files.The complete anonymised data set can be provided upon request.
