# Supplementary material for: Traumatic brain injuries in a district level emergency department in Cape Town: describing patients’ journey from arrival to CT scan and neurosurgery
Source: BMC Emerg Med. 2025 Jul 15;25:123. doi: 10.1186/s12873-025-01277-x (PMC12261671; doi:10.1186/s12873-025-01277-x)
Supplement: Supplementary file 1 — Supplementary Material 1 [file 12873_2025_1277_MOESM1_ESM.docx]

Supplementary material

Box 1: Western Cape Head Injury Guidelines -Indications for Computed Tomography of the brain for patients with Traumatic Brain Injury [39]

| **Within 1 hour** | **Within 8 hours** |
| --- | --- |
| GCS <13 at any point since injury | Amnesia for events more than 30 minutes prior to injury |
| GCS 13 or 14, 2 hours after injury | Dangerous mechanism of injury:   - High-speed road traffic crash - Fall from height (greater than 1 metre) |
| Suspected open/depressed skull fracture |  |
| Any sign of base of skull fracture |  |
| Post-traumatic seizure |  |
| Focal neurological deficit |  |
| 1 or more episode of vomiting since injury |  |
| Penetrating orbital injury |  |
| Tangential GSW head |  |
| Any penetrating head injury |  |
| Loss of consciousness/amnesia and one of the following:   - Age 65 years or older - Coagulopathy - Dangerous mechanism of injury |  |

GCS Glasgow Come Scale GSW Gunshot Wound

Box 2: A description of process time variables of participants’ journey from scene until CT scan or neurosurgery

| **Pre-hospital Process Time Variables** | | **Hospital Process Time Variables** | |
| --- | --- | --- | --- |
| *Response Time* | Time from EMS call received until ambulance arrival at scene | *Door to triage* | Time from arrival at hospital until completion of triage process |
| *Scene Time* | Time from ambulance arrival at scene until departure from scene with patient | *Triage to consultation* | Time from completion of triage process to beginning of clinician consultation |
| *Transportation time* | Time from scene departure until arrival at hospital | *Consultation to ED disposition* | Time from beginning of clinician consultation until final disposition decision is made |
| *Total Mission time* | Total mission time = Time from EMS call received until arrival at hospital with patient | *ED disposition to ED exit* | Time from disposition decision until physical exit from ED |
|  |  | *Total ED length of time* | Total time from arrival at hospital until physical exit from the ED |
|  |  | *Door-to-CT scan* | Time from arrival at ED until completion of CT scan |
|  |  | *Door-to-neurosurgery time* | Time from arrival at ED until neurosurgical operative intervention. |

EMS. Emergency Medical Services ED Emergency Department CT Computed Tomography
